# The impact of peer effect of industrial robot application on enterprise carbon emission reduction

**DOI:** 10.1038/s41598-024-62888-1

**Published:** 2024-05-27

**Authors:** Jinhua Guo, Shuaiwen Chang, Mengnan Guo

**Affiliations:** 1 School of Business Administration, Shanxi University of Finance and Economics, No.140, Wucheng Road, Xiaodian District, Taiyuan, Shanxi China; 2 School of Accounting, Shanxi University of Finance and Economics, Taiyuan, China

**Keywords:** Environmental social sciences, Environmental economics, Environmental impact, Socioeconomic scenarios

## Abstract

The application of intelligent technology, such as industrial robots, is related to the environmental governance effectiveness of enterprises and the realization of the goal of “carbon peak and carbon neutrality”. Due to their similar external environments, driven by economic rationality, peer enterprises will mimic the robotics applications of other enterprises, which in turn will affect the enterprises' carbon emissions. However, little literature has explored the impact of industrial robot application on enterprise carbon emissions from the perspective of peer effect. Based on the data of Shanghai and Shenzhen A-share manufacturing listed enterprises in China from 2011 to 2021, this paper explores the impact of industrial robot application on carbon emission reduction of manufacturing enterprises from the perspective of peer effect. It is found that the industry peer effect and regional peer effect brought by the application of industrial robots are conducive to promoting the carbon emission reduction of enterprises. Among them, the industry peer effect of industrial robot applications promotes carbon emission reduction by enhancing the green innovation ability of enterprises, while the regional peer effect promotes carbon emission reduction by improving the service level of enterprises. It is further found that the degree of industry competition and the level of environmental regulation have inverted U-shaped moderating effects on the industrial robot application industry peer effect, regional peer effect, and enterprises' carbon emission reduction, respectively. The results enrich the research on the impact of industrial robot application on carbon emission reduction of manufacturing enterprises and provide policy implications for improving the environmental performance of enterprises.

## Introduction

Since the reform and opening up, China’s economy has developed rapidly and has now become the second-largest economy in the world. As the backbone of the Chinese economy, the manufacturing industry has played a pivotal role in bolstering economic growth. However, its high energy consumption, pollution, and carbon emissions also pose monumental challenges for China to achieve its carbon peak and neutrality objectives. According to IEA statistics, China’s carbon emissions in 2022 reached 11.477 billion metric tons, accounting for 31.2% of total global emissions, and industrial source carbon dioxide emissions account for around 83% of the total emissions. Overall, China remains the world’s largest carbon emitter. Therefore, promoting the green and low-carbon transformation of economic development is an urgent issue for the Chinese government.

With the rise of a new round of industrial revolution represented by intelligent manufacturing on a global scale, intelligent development is of great significance in achieving industrial structure optimization and promoting green development. The application of industrial robots is an important initiative to promote the intelligent upgrading and green transformation of China’s manufacturing enterprises, and it plays an important role in resource integration, green production, and environmental supervision^1^. According to the International Federation of Robotics’ Global Robotics Report, China’s density of industrial robots in manufacturing reached 322 units per 10,000 employees in 2021, ranking fifth globally. This indicates that intelligent technology represented by industrial robots is progressively becoming the new engine driving China’s economic growth and gradually becoming a key driving force to solve the inherent contradiction between economic growth and carbon emission reduction at the micro-enterprise level.

Peer effect refers to the phenomenon where an individual within a particular group consciously attends to and emulates the behavior of other members when confronted with a similar environmental context, which can be categorized into industry peer effect and regional peer effect. In the era of the digital economy, information sharing and collaboration among enterprises are increasingly strengthened, and at the same time, imitation behavior among enterprises is also intensified. When an enterprise achieves significant economic and environmental benefits in the application of industrial robots, other enterprises in the same industry or the same region tend to follow suit and increase their investment in intelligent technologies such as industrial robots, thus forming a peer effect^2^. For example, target enterprises will tend to obtain relevant information from the green innovation activities of other enterprises in the same industry and imitate them in making decisions. Concurrently, the peer effect of industrial robot application disseminates robotic technologies across more enterprises, thereby fostering an ecological community encompassing production, research and development, and environmental pollution mitigation, synergistically advancing the harmonious coexistence of economic growth and green transition. Therefore, in this context, it is of great significance to explore the impact of the peer effect of industrial robots on the carbon emissions of Chinese manufacturing enterprises, to realize the goal of “carbon peak, carbon neutral” in China. In particular, can the peer effect brought about by the application of industrial robots reduce the carbon emission intensity of focal enterprises? What is the mechanism of its influence? These questions deserve attention but have not been examined in depth in existing studies.

A synthesis of existing literature reveals that the application of artificial intelligence technologies, such as industrial robots, within the manufacturing sector has exerted varying degrees of influence on economic growth, labor remuneration, and employment levels, as well as industrial composition. For example, Du and Lin^3^ found that the application of industrial robots can promote economic growth by increasing total factor productivity. Dekle^4^ found that the application of industrial robots will improve productivity, promote the expansion of enterprises' production scale, and create more jobs. However, there are also studies pointing out that intelligent technologies such as industrial robots may replace low-complexity, high-repetitive jobs, resulting in a crowding-out replacement of labor^5^. Li and Xu^6^ found that AI applications have a facilitating effect on the optimization and upgrading of industrial structure, and are heterogeneous in terms of inter-regional and intra-regional development gaps. Concomitantly, as environmental concerns have gained prominence, scholars have directed their attention toward the reality that the application of industrial robots influences carbon emissions; however, the majority of extant studies primarily concentrate on the macro-regional scale. For example, Tao et al.^7^ found that industrial intelligence reduces regional carbon emission intensity by promoting green technological innovation, and the green technology innovation effect of industrial robot application is heterogeneous among different industries and regions^8^. Meng et al.^9^ found that industrial intelligence can not only reduce the local carbon emission intensity but also reduce the carbon emission intensity of neighboring regions through the spatial spillover effect. Shen and Yang^10^, based on provincial-level panel data in China, found that the introduction and installation of industrial robots by enterprises significantly reduces carbon emissions and the concentration of fine particulate matter in the air, and has a synergistic effect of reducing pollution and carbon. At the micro-level, some scholars have posited that the application of industrial robots facilitates the impetus for enterprises to embrace cleaner production methodologies^1^. Liang et al.^11^ discovered that the application of industrial robots can foster green transformation within enterprises by enhancing their green innovation capabilities. Xu et al.^12^ conducted their research on heavily polluting enterprises and uncovered that industrial intelligence exerts a significant positive influence on the green transformation of enterprises, wherein state-owned enterprises, manufacturing industries, and energy production and supply industries exhibit more pronounced effects.

To summarize, first, current research on industrial robot applications mainly focuses on their economic benefits, while relatively few studies have explored their environmental benefits. Although some scholars have paid attention to the impact of industrial robot applications on carbon emission reduction, these studies are primarily focused at the macro-provincial and municipal levels, lacking research at the micro-enterprise level. Secondly, existing studies on the application of industrial robots and enterprises' carbon reduction have largely ignored the environmental benefits brought about by the imitation or following behavior of enterprises in the same industry or region. However, in the dynamically changing era of the digital economy, there exists a wide range of social interactions such as competition, cooperation, imitation, norms, and pressures among interconnected peer enterprises, which can subconsciously influence enterprises' application of industrial robots and the effects on their carbon emission reductio^8^. Therefore, it is necessary to include peer enterprise behavior in the research scope and explore the impact of the peer effect of industrial robot applications on enterprise carbon emission reduction.

This paper takes the balanced panel data of Chinese A-share listed enterprises in the manufacturing industry from 2011 to 2021 as the research samples, and empirically examines the impact of the peer effect of the application of industrial robots on the carbon emission reduction of the manufacturing enterprises by using the fixed effect model, the mediation effect model and the moderating effect model. The reason for selecting listed enterprises as the research object is that listed enterprises, due to their scale, influence, and industry representativeness, have a demonstration effect in promoting green transformation, and their behavior in carbon emission and pollution control has an important impact on the sustainable development of the whole society. The innovations and marginal contributions of this study are as follows: Firstly, grounded in the strategic ecology theory, this paper investigates the existence of the peer effect stemming from enterprises' application of industrial robots and its subsequent impact on their carbon emission reduction efforts. This enriches the understanding of the pollution control effects associated with industrial robot applications and unravels the driving forces behind enterprises' carbon emission reduction endeavors. Secondly, based on Marshall's externality theory, this paper explores the role of industry peers and regional peers in promoting enterprises' green innovation and service level, providing strategic guidance for enterprises to formulate accurate and efficient green development strategies. Thirdly, based on the industrial organization theory and the environmental Kuznets curve theory, this paper enterprises the degree of industry competition and the level of regional environmental regulation into the research framework and analyzes the regulating effect of the external environment on the peer effect of industrial robot applications and carbon emission reduction of enterprises, and provides certain references for the government to formulate policies.

The remaining chapters of this paper are structured as follows: The second part delves into theoretical analysis and hypothesis formulation. The third part encompasses model specification, variable descriptions, and data source elucidation. The fourth part is the result of the baseline regression and robustness test. The fifth part investigates the mediating effects analysis and moderating effect examination. The sixth part articulates conclusions and further discussion. The final part is the policy implications and research prospects.

## Theoretical analysis and hypothesis formulation

### Industrial robot application peer effect and enterprises carbon emission reduction

#### Industrial robotics application industry peer effect and regional peer effect

Strategic ecology theory investigates the strategic interplay among groups of enterprises and their relationship with environmental dynamics throughout their developmental trajectory. It emanates from the ecosystem constituted by enterprise clusters and their encompassing environments. In this evolutionary process, competitive learning and institutional isomorphism coexist as two states. Competitive learning refers to the self-renewal and collaborative evolution of enterprises within a dynamic competitive landscape, advocating strategic competition and adaptive learning. Conversely, institutional isomorphism alludes to the gradual elimination of misaligned enterprise entities during the evolution of enterprise agglomerations, underscoring institutional constraints and environmental pressures^13^. Grounded in the perspectives of competitive learning and institutional isomorphism under the strategic ecology theory framework, this study explores the peer effect stemming from enterprises' industrial robot application, bifurcating into industry peer effects and regional peer effects.

At the industry level, the strategic ecological theory regards the enterprise group and its external environment as an organic "industrial ecosystem". Due to similar technological environments, market space, etc., enterprises in the same industry have intense competitive relationships^14^. Competitive learning theory points out that enterprises must realize continuous competition and learn through self-renewal and collaborative evolution. On one hand, when some enterprises in the same industry achieve a certain competitive advantage in the application of industrial robots, it will inspire other enterprises to catch up actively and acquire advanced industrial robotics technology to enhance their competitiveness^15^. On the other hand, sharing the successful experience of industrial robot application among enterprises in the same industry helps reduce the risk of investment failure of the focal enterprises and improves their industrial robot application level, which reflects the concept of win–win learning^2^.

At the regional level, institutional isomorphism theory postulates that enterprise clusters exhibit an inclination to conform to the predominant institutional norms and pressures pervasive within their respective environments^16^. On the one hand, the government has a guiding and regulating role in the application of industrial robots in local enterprises. To comply with the local government's policy initiatives and obtain policy support, enterprises in the same region usually actively explore the practice of industrial robotics application, thus forming a peer effect within the region. On the other hand, the strategic choices of enterprises are influenced by the socio-cultural values of the region^17^. The application of industrial robots conforms to the concept of regional green development. Therefore, enterprises in the same region will often choose to increase the application of advanced industrial robotics technology to win a good reputation and stakeholder support, thus forming a regional peer effect.

- Hypothesis ${\text{H}}_{1\text{a}}$: Industrial robot application has an industry peer effect.
- Hypothesis ${\text{H}}_{1\text{b}}$: Industrial robot application has a regional peer effect.

#### Industrial robot application peer effect and enterprises carbon emission reduction

Relying on its versatility and pervasiveness, intelligent technology has fostered new economic forms through deep integration with manufacturing, providing a new impetus for enterprises' green development^18^. In particular, the peer effect of industrial robot applications has played an important role in promoting the green transformation of enterprises and reducing carbon emissions. Firstly, the peer effect of industrial robot application can enhance the external connection and resource acquisition of enterprises, which promotes the knowledge sharing of peer enterprises in product production, resource optimization allocation, and pollution control, effectively improves the resource utilization efficiency of enterprises, and reduces the carbon emission intensity of enterprises^19^. Secondly, the application of industrial robots in the peer enterprises provides decision-making reference for the focus enterprises, and the focus enterprises can benefit from the advanced experience and technology of other enterprises, thus expanding the scope of industrial robot application and reducing the carbon emission intensity of enterprises^20^. Finally, the application of industrial robots in peer enterprises helps to gather advanced production factors such as technology and data in the industry and region. Through introducing intelligent technologies and equipment, enterprises can realize automation and intelligence in the production process, providing a new impetus for carbon emission reduction and sustainable development of focal enterprises.

- Hypothesis ${\text{H}}_{1\text{c}}$: The industry peer effect of industrial robot application is conducive to promoting the carbon emission reduction of focus enterprises.
- Hypothesis ${\text{H}}_{1\text{d}}$: The regional peer effect of industrial robot application is conducive to promoting the carbon emission reduction of focus enterprises.

### The mediating effects of green innovation capacity and the level of servitization

Marshall's externality theory points out that the agglomeration of enterprises can produce an externality economy, and divide it into technology externality and market externality^21^. Technology externality manifests as the spillover effects of knowledge agglomeration and technological proximity on enterprise technology innovation, while market externality emphasizes the adjacency effects of economic linkages and geographic proximity on the division of labor among enterprises. Therefore, this paper considers that there may be differences in the path of carbon emission reduction driven by industry peer effect and regional peer effect of industrial robot application, and attempts to analyze it from two aspects: green innovation and service-oriented transformation.

Firstly, the industry peer effect of industrial robot applications reduces enterprise carbon emissions by promoting green innovation. Marshall emphasized that enterprise clustering facilitates knowledge spillover and technology diffusion, thus generating technology externality^21^. As enterprises in the same industry possess similar technologies, knowledge, and innovation experience, the industry peer effect accelerates the circulation and sharing of these resources within the cluster. Consequently, enterprises can obtain external knowledge and innovation resources through more channels, enhancing their green innovation capabilities. Simultaneously, the peer effect in the industry strengthens the interaction among subjects of innovation, motivating enterprises’ green innovation activities by sharing risks and saving innovation costs. As pointed out by Wu et al.^22^, the peer effect in the industry reduces the green innovation costs and risks for enterprises, thereby improving their ability for green innovation and significantly enhancing their performance in environmental pollution prevention and environmental governance.

Secondly, the regional peer effect of industrial robot applications reduces enterprise carbon emissions by enhancing the level of servitization. Marshall noted that enterprise agglomeration could bring about economies of scale in factor markets such as labor and intermediate goods, thereby generating market externality^21^. The regional peer effect promotes the exchange and interaction of experience and knowledge among enterprises in the same region, which is conducive to providing diversified and customized solutions for customers, thus enhancing the level of servitization^23^. At the same time, the regional peer effect reduces the degree of information asymmetry between the supply and demand sides. This effectively reduces service costs and improves service efficiency for enterprises. With the improvement of the level of servitization, enterprises achieve higher energy utilization efficiency by introducing advanced technologies, improving product design, and optimizing production processes. For example, the outsourcing business promoted by servitization in the manufacturing industry can carry out a disruptive reform of the production system, which is conducive to reducing energy consumption and carbon emission intensity in the production process^24^. Based on the above analysis, this paper proposes the following hypotheses:

- Hypothesis ${\text{H}}_{2\text{a}}$: Green innovation capability mediates the relationship between the industry peer effect of industrial robot applications and corporate carbon emission reductions.
- Hypothesis ${\text{H}}_{2\text{b}}$: The level of servitization mediates the relationship between the regional peer effect of industrial robot applications and corporate carbon emission reductions.

### The moderating effects of the degree of industry competition and the level of regional environmental regulation

The effect of industry and regional peer influence from industrial robot application on enterprise carbon emission reduction is likely to be moderated by external factors. Based on the theory of industrial organization and the environmental Kuznets curve, this paper studies the regulating effect of industrial competition and regional environmental regulation on the relationship between them.

Firstly, the influence of the industry peer effect of industrial robot application on enterprise carbon emission reduction is regulated by the degree of industry competition. According to the industrial organization theory, a moderate degree of industry competition is conducive to technological innovation and knowledge diffusion within the industry^25^. When competition is insufficient, enterprises face little market pressure, lack intrinsic motivation for green innovation, and may neglect environmental issues in pursuit of economic gains from robotics^2^. Conversely, excessive competition intensifies resource contention, impeding technical cooperation and knowledge sharing, thus hindering the industry's peer effects. However, in a moderately competitive environment, the industry peer effect of enterprise industrial robot applications promotes the diffusion of green technology and strengthens innovation cooperation, thus promoting the low-carbon transformation of the entire industry.

Secondly, the impact of the regional peer effect of industrial robots on corporate carbon emission reduction is moderated by the level of environmental regulation. The environmental Kuznets curve theory points out that when environmental regulation is too lax, enterprises lack external regulatory pressure, so enterprises may ignore the importance of environmental pollution prevention and control^26^. Conversely, when environmental regulations are too strict, although the policy may force enterprises to increase investment in energy conservation and emission reduction, excessive compliance costs may also hinder the application and diffusion of new technologies such as industrial robots^27^. However, a moderate level of environmental regulation can effectively stimulate enterprises' environmental awareness and green innovation power. At the same time, in this context, enterprises can actively learn advanced environmental protection experience based on regional peer effect and actively apply green environmental protection technology to reduce carbon emissions^28^. Based on this analysis, the following hypotheses are proposed:

- Hypothesis ${\text{H}}_{3\text{a}}$: The degree of industry competition plays an inverted U-shaped regulating role in the relationship between industrial robot application industry peer effect and enterprise carbon emission reduction.
- Hypothesis ${\text{H}}_{3\text{b}}$: The regional environmental regulation level plays an inverted U-shaped regulating role in the relationship between the regional peer effect of industrial robots and the carbon emission reduction of enterprises.

## Research design

### Model specification

In order to test whether there is a peer effect in the application of industrial robots in enterprises, this paper draws on Huo et al.^2^ and constructs the following model for testing:

1 $$lnexposure_{e,t} = \alpha_{0} + \alpha_{1} lnexposure\_mean_{e,t} + \alpha_{2} X_{e,t} + \mu_{e} + \omega_{t} + \epsilon_{e,t}$$

In order to test the impact of the peer effect of industrial robot application on the carbon dioxide emission intensity of the focal enterprise, the benchmark model is set as follows:

2 $$lnco2_{e,t} = \alpha_{0} + \alpha_{1} lnexposure\_mean_{e,t} + \alpha_{2} X_{e,t} + \mu_{e} + \omega_{t} + \epsilon_{e,t}$$

In model (1), the dependent variable is ${\text{lnexposure}}_{e,t}$, denoting the penetration degree of industrial robots in the focal enterprises; the dependent variable in the model (2) is $lnco2_{e,t}$, which represents the intensity of CO2 emissions of enterprises; the core explanatory variable is $lnexposure\_mean_{e,t}$, which contains the industry peer effect ($lnexposure\_mean1_{e,t}$) and the regional peer effect ($lnexposure\_mean2_{e,t}$) of industrial robot application*.* $X_{e,t}$ denotes a collection of control variables. $\mu_{e} ,\omega_{t}$ denotes control for enterprises’ fixed effects and time fixed effects, respectively; $\epsilon_{e,t}$ is a randomized disturbance term.

### Variable descriptions

#### Dependent variable

Carbon emission intensity (*lnco2*). This paper measures the carbon emission intensity of an enterprise by the ratio of the enterprise's CO2 emissions to its operating revenue. Specifically, enterprises' CO2 emission data are obtained through manual collation based on annual reports of listed enterprises, enterprises' social responsibility reports, and environmental reports. The Greenhouse Gas Protocol, compiled by the World Business Council for Sustainable Development (WBCSD) and the World Resources Institute (WRI), categorizes enterprises carbon emissions into three scopes: Scope 1, direct greenhouse gas emissions primarily from the production processes; Scope 2, indirect emissions from purchased electricity and heat; and Scope 3, other indirect emissions. Following Wang et al.^29^, this study adopts the sum of Scope 1 and Scope 2 emissions as an enterprise's total carbon emissions. For enterprises that do not directly report carbon emissions but disclose energy consumption, electricity usage, and heat consumption, we calculate their direct (Scope 1) and indirect (Scope 2) emissions separately according to the Guidelines for Accounting and Reporting Greenhouse Gas Emissions from Enterprises issued by China's National Development and Reform Commission and then sum them to obtain the enterprise's total carbon emissions.

#### Independent variable

The industry peer effect (*lnexposure_mean1*) and regional peer effect (*lnexposure_mean2*) of industrial robot application. Following the approach of Acemoglu et al.^30^, this paper adopts enterprise-level robot penetration to measure industrial robot application, and the specific measurement methods are as follows:

*Step 1* Calculate the robot installation intensity of each industry in China.

$$density_{it}^{CH} = \frac{{robot_{it}^{CH} }}{{labour_{i,t = 2010}^{CH} }}$$

where $robot_{it}^{CH}$ denotes the stock of industrial robots in industry* i* in year *t* in China, $labour_{i,t = 2010}^{CH}$ denotes employment in industry *i* in the base year 2010 in China, and $density_{it}^{CH}$ denotes the robot installation intensity of industry *i* in year *t* in China.

*Step 2* Construct the penetration index of industrial robots at the enterprise level in China.

$$exposure_{e,i,t} = \frac{{pwp_{e,i,t = 2011} }}{{manupwp_{e,i,t = 2011} }} \times density_{it}^{CH}$$

where $pwp_{e,i,t = 2011}$ denotes the share of production workers in enterprises *e* of industry *i* in the base year 2011; $manupwp_{e,i,t = 2011}$ is the median share of production workers across all manufacturing enterprises in 2011; $exposure_{e,i,t}$ denotes the industrial robot penetration of enterprise *e* in industry* i* in year *t*. In this paper, the logarithmic form of industrial robot penetration of enterprise *e* in China in year *t* is taken as the core explanatory variable, represented by $lnexposure_{e,t}$.

*Step 3* Construct the industrial robot application index of the peer enterprises in the industry and region.

With reference to Grennan^31^, enterprises in the same industry or registered city as the focal enterprise are defined as industry peers or regional peers, respectively. The average level of robot application among these peer enterprises is utilized to measure industry peer robot application *(lnexposure_mean1)* and regional peer robot application* (lnexposure_mean2).*

#### Control variables

Referring to the studies by Liang et al.^11^, Nie et al.^32^, and Xie et al.^33^, the following control variables are selected: employee size (*lnlabour*), measured by the logarithmic number of employees; Enterprise size (*lnsize*), measured by the logarithm of total assets; Enterprise age (*lnage*), measured by the logarithm of the number of years since the establishment; Cash flow ratio (*cashflow*), the ratio of net cash flow from operating activities to current liabilities; Profitability (*roa*), the ratio of net profit to total assets; Ownership nature (*soe*), a dummy variable that takes the value of 1 for state-owned enterprises and 0 for non-state-owned enterprises. This paper selects the above control variables mainly because they are important factors affecting the carbon emission reduction of enterprises. Generally speaking, the greater the number of employees, the more likely it will lead to an increase in additional energy consumption and carbon emissions of the enterprise, such as employee commuting and office electricity usage. Enterprises with larger asset sizes tend to exhibit increasing returns to scale in production and decreasing returns to scale in costs, resulting in relatively lower carbon emission intensities. The older the enterprise, the greater the difficulty in green transformation and upgrading, and this type of enterprise is likely to have higher carbon emission intensities. The cash flow ratio reflects the cash flow status and debt-paying ability of enterprises. Enterprises with higher cash flow ratios can invest more funds in energy conservation, emission reduction, and environmental protection transformation, thus reducing carbon emission intensity. In China, most enterprises mainly rely on extensive development to improve their profits, and such enterprises tend to have high carbon emission intensity. Enterprises with different property rights may also have large differences in carbon emissions due to differences in government regulation.

### Data sources

This study takes the data of China's listed manufacturing enterprises on the Shanghai and Shenzhen A-share markets from 2011 to 2021 as the overall sample. Enterprises' financial data is obtained from the CSMAR and WIND databases. Carbon emissions data is sourced from listed enterprises' annual reports, enterprises’ social responsibility reports, and environmental reports. Employment data for China's manufacturing subsectors comes from the China Industrial Statistical Yearbook, while U.S. subsector employment data is from the NBER-CES database. With reference to Wang and Dong^34^, based on GB/T4754-2011 National Economic Industry Classification and Code and International Standard Industry Classification of All Economic Activities (Fourth edition), this paper matches the major industries belonging to listed manufacturing enterprises with the industrial robot data published by the International Federation of Robotics. To mitigate potential biases, the sample is treated as follows: (1) enterprises under special treatment such as ST and PT are excluded; (2) observations with missing relevant data are removed; (3) enterprises without consecutively published annual reports are omitted. Moreover, to alleviate the influence of outliers, continuous variables are winsorized at the 1st and 99th percentiles. Descriptive statistics for the main variables are reported in Table 1.

**Table 1:** Descriptive statistics of main variables.

Variable	N	Mean	SD	Min	Max
lnco2	7267	2.461	1.551	− 6.471	6.345
lnexposure	7267	5.724	0.316	2.923	7.849
lnexposure_mean1	7267	2.436	1.499	− 5.782	6.039
lnexposure_mean2	7267	2.554	0.950	− 1.920	6.105
lnlabour	7267	7.802	1.0527	4.3567	12.118
lnsize	7267	22.019	1.083	19.082	27.307
lnage	7267	2.720	0.403	0.693	3.951
cashflow	7267	0.049	0.067	− 0.407	0.488
roa	7267	0.042	0.065	− 0.567	0.351
soe	7267	0.309	0.462	0.000	1.000

## Analysis of empirical results

### Baseline regression

The test results of the peer effect in industrial robot applications are presented in columns (1) and (2) of Table 2. In column (1), the regression coefficient of industrial robot application in the peer enterprises in the industry is positive and statistically significant at the 1% level. In column (2), the regression coefficient of industrial robot application in the peer enterprises in the region is positive and significant at a 1% level. The research results show that the application of industrial robots in enterprises has a significant peer effect, that is, the application of industrial robots in focus enterprises shows a growing trend with the improvement of the application degree of industrial robots in the peer enterprises in the industry and region. The research hypothesis ${\text{H}}_{1\text{a}}$ and ${\text{H}}_{1\text{b}}$ of this paper are valid.

**Table 2:** Baseline regression results.

Variables	(1)	(2)	(3)	(4)	(5)
	lnexposure	lnexposure	lnco2	lnco2	lnco2
lnexposure			− 0.035***		
			(0.010)		
lnexposure_mean1	0.942***			− 0.033***	
	(0.005)			(0.010)	
lnexposure_mean2		0.416***			− 0.026**
		(0.035)			(0.011)
lnlabour	− 0.009	− 0.189***	− 0.001	− 0.001	0.003
	(0.007)	(0.052)	(0.026)	(0.026)	(0.032)
lnsize	0.015*	0.263***	− 0.031	− 0.032	− 0.029
	(0.008)	(0.056)	(0.027)	(0.027)	(0.029)
lnage	0.013	2.000***	0.039	0.033	− 0.012
	(0.019)	(0.171)	(0.057)	(0.057)	(0.063)
cashflow	0.009	− 0.228	0.461***	0.461***	0.415***
	(0.017)	(0.161)	(0.105)	(0.105)	(0.099)
roa	0.027	− 0.560***	− 0.601***	− 0.601***	− 0.512***
	(0.025)	(0.196)	(0.093)	(0.093)	(0.102)
soe	− 0.003	0.082	0.088*	0.088*	0.061
	(0.013)	(0.102)	(0.051)	(0.051)	(0.054)
_cons	− 0.297**	− 8.351***	6.375***	6.406***	6.411***
	(0.140)	(0.932)	(0.470)	(0.467)	(0.452)
Obs	7267	7267	7267	7267	7267
Firm/year	Yes	Yes	Yes	Yes	Yes
R-squared	0.898	0.799	0.041	0.040	0.033

***, **, * denote significance levels at 1%, 5%, and 10%, respectively, with robust standard errors in parentheses.

The test results of the impact of peer effect on carbon emission reduction by industrial robot application are presented in columns (3)–(5) of Table 2. Column (3) shows that for every 1% increase in the penetration of industrial robots, the carbon emission intensity decreases by 0.035%, which indicates that the application of industrial robots can prompt enterprises to adopt more intensive production models, thereby reducing carbon emission intensity. In column (4) of Table 2, the regression coefficient of industrial robot application in industrial peer enterprises is − 0.033, which is significant at the 1% level, indicating that the improvement of industrial robot application level in industrial peer enterprises is conducive to the reduction of carbon emission intensity. In column (5), the regression coefficient of industrial robot application in regional peer enterprises is − 0.026, and is significant at the 5% level, indicating that the increase in the application level of industrial robots in regional peer enterprises is conducive to reducing the carbon emission intensity of enterprises. Hypotheses ${\text{H}}_{1\text{c}}$ and ${\text{H}}_{1\text{d}}$ are tested.

### Robustness test

#### Endogeneity issues

In this paper, referring to the research of Leary and Roberts^35^, the average annual stock characteristic return (iv1) of peer enterprises in the industry and the average annual stock characteristic return (iv2) of peer enterprises in the region are adopted as instrumental variables. This is because, on the one hand, economic activities and technological choices among enterprises within an industry or region tend to converge due to similarities in market demand and policy environment. The mean stock-specific returns can reflect the economic environment or technological progress trend in the industry, which potentially correlates with enterprises' industrial robot application^35^. On the other hand, the mean stock-specific returns of peer enterprises in the same industry or region reflect the comprehensive performance of enterprises' economic activities, yet it is not directly related to enterprises' carbon emissions. Therefore, this instrumental variable satisfies the exogeneity condition. As presented in Table 3, the first-stage regression results reject the "weak instrumental variable" hypothesis based on the F-test, and the significant LM statistic suggests that the model rejects the hypothesis of "under-identification of instrumental variables." In the second-stage regression results, the regression coefficients of the core explanatory variables are significantly negative at the 1% level, indicating that the peer effect of industrial robot applications is conducive to promoting enterprise carbon emission reduction. The robustness of the conclusions of this paper is verified.

**Table 3:** Endogeneity issues.

Variables	iv1		iv2	
	Firsstage	Secondstage	Firsstage	Secondstage
	(1)	(2)	(3)	(4)
lnexposure_mean1	0.736***	− 0.050***		
	(0.014)	(0.016)		
lnexposure_mean2			0.665***	− 0.047***
			(0.010)	(0.016)
lnlabour	− 0.066***	0.004	− 0.032**	0.010
	(0.018)	(0.015)	(0.016)	(0.016)
lnsize	0.117***	− 0.039**	0.069***	− 0.033*
	(0.019)	(0.015)	(0.017)	(0.017)
lnage	0.378***	− 0.029	0.180***	− 0.059
	(0.065)	(0.053)	(0.056)	(0.057)
cashflow	− 0.188**	0.444***	0.108	0.426***
	(0.083)	(0.067)	(0.074)	(0.076)
roa	− 0.459***	− 0.583***	− 0.215***	-0.503***
	(0.089)	(0.072)	(0.080)	(0.082)
soe	0.117***	0.085***	0.132***	0.051
	(0.041)	(0.033)	(0.038)	(0.038)
_cons	− 3.659***	6.321***	− 3.701***	6.201***
	(0.323)	(0.285)	(0.306)	(0.308)
Obs	7267	7267	7267	7267
Firm/year	Yes	Yes	Yes	Yes
LM	1865.147 [0.0000]		2271.787 [0.0000]	
F	2650.336 [16.380]		4161.407 [16.380]	

***, **, and * indicate significance levels of 1%, 5%, and 10%, respectively, with robust standard errors in brackets.

#### Eliminate policy interference

During the sample period, the Chinese government introduced the 'carbon emission trading' policy, which may have an impact on the estimation results. Referring to Han and Li^36^, this paper further added the dummy variable (*cbmarket)* of the "carbon emission trading right" policy to control the impact of exogenous policies on the estimation results. From the regression results in columns (1)–(2) of Table 4, after adding the variable *cbmarket*, the core explanatory variable is still significantly negative, indicating that the conclusion of this paper is robust.

**Table 4:** Robustness test.

Variables	(1)	(2)	(3)	(4)	(5)	(6)
	lnco2	lnco2	lnco2	lnco2	lnco2	lnco2
lnexposure_mean1	− 0.033***		− 0.038**		− 0.029***	
	(0.010)		(0.018)		(0.011)	
lnexposure_mean2		− 0.027**		− 0.032**		− 0.025**
		(0.011)		(0.016)		(0.011)
cbmarket	0.013	0.008				
	(0.017)	(0.018)				
lnlabour	− 0.001	0.004	− 0.009	− 0.009	− 0.001	− 0.005
	(0.026)	(0.032)	(0.026)	(0.027)	(0.027)	(0.033)
lnsize	− 0.032	− 0.028	− 0.100***	− 0.090***	− 0.040	− 0.024
	(0.027)	(0.029)	(0.035)	(0.029)	(0.029)	(0.031)
lnage	0.033	− 0.011	0.078	0.072	0.030	− 0.015
	(0.057)	(0.063)	(0.076)	(0.080)	(0.059)	(0.063)
cashflow	0.461***	0.416***	0.387***	0.337***	0.444***	0.388***
	(0.105)	(0.099)	(0.132)	(0.117)	(0.106)	(0.099)
roa	− 0.601***	− 0.512***	− 0.460***	− 0.411***	− 0.604***	− 0.503***
	(0.093)	(0.102)	(0.141)	(0.151)	(0.091)	(0.102)
soe	0.088*	0.061	0.027	-0.015	0.107**	0.085*
	(0.051)	(0.054)	(0.070)	(0.097)	(0.050)	(0.049)
_cons	6.406***	6.403***	7.836***	7.662***	6.573***	6.315***
	(0.467)	(0.452)	(0.708)	(0.538)	(0.537)	(0.548)
Obs	7267	7267	4140	4140	7267	7267
Firm/year	Yes	Yes	Yes	Yes	Yes	Yes
Region/industrial	No	No	No	No	Yes	Yes
R-squared	0.040	0.033	0.047	0.044	0.056	0.045

***, **, and * indicate significance levels of 1%, 5%, and 10%, respectively, with robust standard errors in brackets.

#### Change sample period

Referring to Du et al.^37^, considering China's new national economic industry classification in 2017, this paper's sample period is adjusted to 2011–2016 for regression analysis. As shown in columns (3)–(4) of Table 4, the coefficients of industrial robot application's industry and regional peer effects are significantly negative at the 5% level, indicating that the negative relationship between the peer effect of industrial robot application and enterprise carbon intensity still holds.

#### Increase fixed effects

To mitigate heterogeneity across industries and regions, referring to Han and Li^36^, based on controlling the fixed effect of enterprises and time, the fixed effect of industry and region is added to the model in this paper. The regression results in columns (5) and (6) of Table 4 show that the coefficients of industrial robot application's peer effect remain significantly negative at different significance levels, further corroborating the robust conclusions.

## Mechanism tests

### Mediating effects test

The theoretical analysis suggests that the industry and regional peer effects of industrial robot application can promote carbon emission reduction in enterprises by improving their green innovation capability and service level, respectively. To test the mediating effect, in this paper further constructs models (3)–(4) based on model (2), and the regression results are shown in Table 5.

3 $$med_{e,t} = \beta_{0} + \beta_{1} lnexposure\_mean_{e,t} + \beta_{2} X_{e,t} + \mu_{e} + \omega_{t} + \epsilon_{e,t}$$

4 $$lnco2_{e,t} = \beta_{0} + \beta_{1} med_{e,t} + \beta_{2} lnexposure\_mean_{e,t} + \beta_{3} X_{e,t} + \mu_{e} + \omega_{t} + \epsilon_{e,t}$$

where *med* denotes the mediating variables: *med1*, the natural logarithm of enterprises’ total green patent applications, capturing its green innovation capability; and *med2*, the ratio of service revenue to operating revenue, measuring the firm’s level of servitization. The remaining variables are consistent with the previous definitions.

**Table 5:** Mediating effects test.

Variables	(1)	(2)	(3)	(4)
	med1	lnco2	med2	lnco2
lnexposure_mean1	0.051***	− 0.032***		
	(0.019)	(0.010)		
med1		− 0.019*		
		(0.010)		
lnexposure_mean2			0.008***	− 0.022*
			(0.002)	(0.012)
med2				− 0.268**
				(0.125)
lnlabour	0.102***	0.001	− 0.002	− 0.002
	(0.036)	(0.026)	(0.005)	(0.035)
lnsize	0.116***	− 0.030	− 0.001	− 0.032
	(0.042)	(0.027)	(0.005)	(0.031)
lnage	− 0.001	0.033	0.020	− 0.000
	(0.098)	(0.057)	(0.013)	(0.065)
cashflow	0.147	0.464***	− 0.019	0.434***
	(0.160)	(0.105)	(0.019)	(0.100)
roa	0.285*	− 0.595***	− 0.055***	− 0.492***
	(0.158)	(0.093)	(0.021)	(0.109)
soe	− 0.090	0.087*	0.001	0.101*
	(0.093)	(0.051)	(0.007)	(0.054)
_cons	− 3.186***	6.345***	0.015	6.488***
	(0.799)	(0.459)	(0.079)	(0.488)
Obs	7267	7267	7267	7267
Firm/year	Yes	Yes	Yes	Yes
R-squared	0.051	0.042	0.056	0.035

***, **, and * indicate significance levels of 1%, 5%, and 10%, respectively, with robust standard errors in brackets.

The result of the mediating effect of enterprises' green innovation capability is presented in columns (1) and (2) of Table 5. In Column (1), the coefficient on industry peer effects is 0.051 and significant at the 1% level, indicating that industry peer effects enhance the green innovation level of enterprises. In Column (2), the coefficient on green innovation is − 0.032 and significant at the 1% level, while the coefficient on industry peer effects is significantly negative at the 10% level. This demonstrates that the industry peer effect of industrial robot application reduces carbon emission intensity by improving the green innovation capability of enterprises, thus validating the hypothesis ${\text{H}}_{{2{\text{a}}}}$.

The results of the mediating effect of the enterprise servitization level are presented in columns (3) and (4) of Table 5. In Column (3), the coefficient on regional peer effects is 0.008 and significant at the 1% level, implying that regional peer effects improve the level of enterprise servitization. In Column (4), the coefficients on both servitization and regional peer effects are significantly negative, indicating that the regional peer effects of industrial robot application facilitate enterprise carbon emission reduction by increasing the level of servitization, thus validating the hypothesis ${\text{H}}_{{2{\text{b}}}}$.

### Moderating effects test

To test the moderating effect of the degree of industry competition, referring to Huo et al.^2^, this paper incorporates the Herfindahl index, the quadratic term of the Herfindahl index, and its interaction term with the industry peer effect into the regression model. The Herfindahl index (*hhi*) measures the degree of market competition. The regression results are shown in column (1) of Table 6. The interaction coefficient of the quadratic term is − 0.139 and is significant at the 5% level. The result indicates that the degree of industry competition has an inverted U-shaped moderating effect on the relationship between the industry peer effect of industrial robot application and carbon reduction. That is, when the degree of industry competition is moderate, the industry peer effect of industrial robot application has the best effect on carbon reduction. However, a too-low degree of competition may lead to a lack of motivation for green innovation in enterprises, while a too-high degree of competition may result in overconsumption of resources or excessive pursuit of short-term interests, weakening the carbon emission reduction effect of the industry peer effect of industrial robot application. The hypothesis ${\text{H}}_{{3{\text{a}}}}$ is supported.

**Table 6:** Moderating effects test.

	(1)	(2)
Variables	lnco2	lnco2
hhi	0.239	
	(0.485)	
hhi * hhi	− 0.024	
	(1.445)	
lnexposure_mean1	− 0.040***	
	(0.010)	
hhi * lnexposure_mean1	0.566***	
	(0.182)	
hhi * hhi * lnexposure_mean1	− 0.139**	
	(0.058)	
er		0.003
		(0.008)
er * er		− 0.588
		(0.582)
lnexposure_mean2		− 0.030***
		(0.011)
er * lnexposure_mean2		− 3.366
		(6.009)
er * er * lnexposure_mean2		− 0.663*
		(0.354)
lnlabour	0.002	0.002
	(0.026)	(0.032)
lnsize	− 0.033	− 0.028
	(0.027)	(0.030)
lnage	0.047	− 0.019
	(0.057)	(0.062)
cashflow	0.463***	0.423***
	(0.105)	(0.099)
roa	− 0.602***	− 0.503***
	(0.094)	(0.101)
soe	0.084*	0.059
	(0.051)	(0.053)
_cons	6.350***	6.442***
	(0.468)	(0.456)
Obs	7267	7267
Firm/year	Yes	Yes
R-squared	0.043	0.036

***, **, and * indicate significance levels of 1%, 5%, and 10%, respectively, with robust standard errors in brackets.

To test the moderating effect of regional environmental regulation, referring to Liu et al.^38^, this paper uses the ratio of the investment in pollution control of exhaust gas and wastewater to the industrial output value in the year of the listed company's location to measure the level of environmental regulation (*er*). The paper incorporates the level of regional environmental regulation, the quadratic term of the level of regional environmental regulation, and its interaction term with the regional peer effect into the regression model. The regression result is shown in column (2) of Table 6. The interaction coefficient of the quadratic term is − 0.663 and is significant at the 10% level. The result demonstrates that the degree of regional environmental regulation has an inverted U-shaped moderating effect on the relationship between the regional peer effect of industrial robot application and carbon reduction. That is, as the level of regional environmental regulation rises, the carbon emission reduction effect of the regional peer effect of industrial robot application is first enhanced and then weakened. The hypothesis ${\text{H}}_{{3{\text{b}}}}$ is supported.

## Conclusions and further discussion

Taking Chinese manufacturing enterprises as the research object, this study systematically investigates the impact of the peer effect from industrial robot application on enterprise carbon emission reduction, and draws the following conclusions:

First, from the perspective of strategic ecology theory, this study found that industrial robot application in manufacturing enterprises has significant industry peer effect and regional peer effect, and both the two peer effects can promote the carbon reduction of enterprises. This conclusion is still valid after various robustness tests, such as endogenous problem treatment, excluding policy interference, and changing the sample size. As one of the advanced energy-saving and emission-reducing technologies, the large-scale application of industrial robots can significantly improve energy utilization efficiency and reduce enterprise carbon emissions. Concomitantly, from the viewpoint of technology diffusion and externality, the industry and regional peer effects accelerate the dissemination of industrial robotics within the manufacturing sector, enabling more enterprises to reap the benefits of energy saving and emission reduction. This study enriches the research scope of the relationship between industrial automation technology and environmental governance effects and also provides theoretical support for policymakers to promote the green transformation of the manufacturing industry.

Secondly, based on the Marshall's externality theory, this study analyzes the paths of industry peer effect and regional peer effect affecting carbon emission reduction of enterprises from the levels of green innovation and servitization level of enterprises. The study finds that positive interactions among enterprises in the same industry, such as technology spillovers, learning demonstrations, and collaborative research and development, facilitate the diffusion and innovative application of advanced energy-saving and emission-reducing technologies within the industry, which is conducive to improving enterprises' green innovation capabilities and thereby promoting carbon emission reduction. The regional peer effect fosters specialized division of labor and cooperation among enterprises in the region, drives the development of productive service industries, and provides robust support for the green and low-carbon transformation of enterprises. This finding provides theoretical guidance for enterprises to formulate green development strategies.

Thirdly, based on industrial organization theory and environmental Kuznets curve theory, this study incorporates the degree of industry competition and the level of regional environmental regulation into the research framework to analyze their moderating effects on the relationship between the industrial robot peer effect and enterprise carbon emission reduction. The results indicate that moderate industry competition promotes enterprises to share green innovation outcomes, generating technology spillovers and learning effects, thereby facilitating the diffusion of energy-saving and emission-reducing technologies within the same industry. Moderate environmental regulation exerts pressure on enterprises to reduce emissions, prompting them to increase environmental protection investment and introduce advanced energy-saving technologies, thus fostering the green and low-carbon transformation of enterprises. This finding suggests that the combination of moderate industrial competition and environmental regulation policies can create favorable external conditions for carbon emission reduction, which provides a basis for the development of differentiated industrial robot promotion and environmental regulation policies in different industries and regions.

In sum up, this study is of great significance both in theory and practice. At the theoretical level, this study has enriched the theoretical research on the correlation between industrial robots and enterprises' carbon emission reduction from the aspects of peer effect driving path, transmission mechanism, and external environmental regulatory factors. At the practical level, this study provides policy implications for the green low-carbon transformation of manufacturing enterprises.

## Policy implications and research prospects

Based on the research findings, the following policy insights are drawn:

First, the peer effect of industrial robot applications should be fully leveraged to promote carbon emission reduction in manufacturing enterprises. On the one hand, enterprises in the same industry or region can explore establishing "robot application alliances" to strengthen experience-sharing initiatives. At the same time, the government should actively promote the incentive mechanism of “industrial robots + carbon emission trading rights” and give certain free carbon emission quota incentives to manufacturing enterprises that adopt industrial robots. On the other hand, the government needs to formulate technical specifications for the industry and promote the construction of regional low-carbon demonstration parks. For example, by building low-carbon demonstration parks in various regions, the government should encourage and guide local enterprises to engage in green innovation cooperation and apply low-carbon technologies, such as industrial robots.

Secondly, enterprises should give full play to the role of green innovation and service-oriented transformation in promoting carbon emission reduction. On the one hand, the government can establish a specialized green innovation fund to provide financial support through equity investment and other means to qualified green innovative manufacturers. At the same time, the government should encourage qualified manufacturing enterprises to establish green innovation laboratories independently and actively explore new models of deep cooperation between industry, academia, and research institutes. On the other hand, enterprises should promote the application of industrial robot technology in all aspects of production and operation, establish a new model of "service-oriented manufacturing", and promote the service-oriented transformation and green and low-carbon development of manufacturing enterprises.

Thirdly, all regions should give full play to the positive role of industrial competition and environmental regulation on carbon emission reduction of enterprises. For manufacturing industry competition policies, the government should establish fair and orderly competition supervision and anti-monopoly rules to create a favorable competitive environment. In terms of regional environmental regulation, the government should establish a dynamic coupling adjustment mechanism between the application of industrial robots and environmental regulations. As the prevalence of robots increases in enterprises, the government needs to evaluate their effectiveness in improving resource utilization and reducing pollutant emissions promptly, adjust environmental regulation standards dynamically, and achieve coordinated unification of economic development and ecological environmental protection.

However, this study has the following research limitations. Firstly, considering that fewer enterprises voluntarily disclose pollution data in their annual reports, this paper only obtained 7267 observations from a sample of listed manufacturing enterprises, which makes it difficult to reflect the full picture of manufacturing enterprises. In future studies, we will consider manually collecting data related to environmental information disclosure of micro, small, and medium-sized enterprises (MSMEs) or more listed enterprises, to more comprehensively and accurately assess the impact on carbon emission reduction of enterprises and expand the universality of research conclusions. Secondly, this study lacks sufficient exploration of the differences in the impact of industrial robot applications on carbon emission reduction across different industries. Future research should attempt to further explore the differences in carbon emission reduction effects of industrial robot applications in different industries (such as high-tech industries and traditional manufacturing industries) through cross-industry comparison.

## Data Availability

Some or all data, models, or codes generated or used during the study are available from the corresponding author by request.
